# The Conservation Implications of the Gut Microbiome for Protecting the Critically Endangered Gray Snub-Nosed Monkey (*Rhinopithecus brelichi*)

**DOI:** 10.3390/ani14131917

**Published:** 2024-06-28

**Authors:** Yanqing Guo, Paul A. Garber, Yijun Yang, Siwei Wang, Jiang Zhou

**Affiliations:** 1 College of Life Sciences, Northwest University, Xi’an 710072, China; 13638111874@163.com (Y.G.); yijunyangyijunyang@gmail.com (Y.Y.); 2 Program in Ecology, Evolution and Conservation Biology, Department of Anthropology, University of Illinois, Urbana, IL 61801, USA; p-garber@illinois.edu; 3 International Centre of Biodiversity and Primate Conservation, Dali University, Dali 671000, China; 4 School of Karst Science, Guizhou Normal University, Guiyang 550003, China

**Keywords:** gut microbiome, seasonality, nutrition, endangered primate, *Rhinopithecus brelichi*

## Abstract

**Simple Summary:**

The last remaining population of gray snub-nosed monkeys (*Rhinopithecus brelichi*) inhabits a small area of fragmented forest within the Fanjinshan National Reserve. As colobine monkeys, these primates are characterized by a highly specialized and elongated, sacculated stomach that maintains a pH between 6 and 7 and a highly diverse microbiota. The colobine gut microbiota exerts a pivotal role in the biochemical conversion of cellulose into short-chain fatty acids, thereby facilitating the host’s energy acquisition. Recognizing the gray snub-nosed monkey’s diet varies seasonally, the adaptation of the gut microbiota to these fluctuations in food resources is imperative for their survival, but little is known about the adaptive responses of the gut microbiome to seasonal variations in food sources and availability. In this study, we provided insights into the adaptations of gut microbiota to seasonal fluctuations in nutrient intake and energy balances for the critically endangered gray snub-nosed monkey. Moreover, we also identified *Pseudomonas* in two samples; the presence of potential pathogens within the gut could pose a risk to other troop members. These findings shed light on the adaptations of the gut microbiota to seasonal fluctuations in immunity and indicate the necessity of a conservation plan that focuses on vegetation and implements measures to prevent disease transmission for this critically endangered species.

**Abstract:**

The gut microbiota plays a crucial role in regulating energy metabolism, facilitating nutrient absorption, and supporting immune function, thereby assisting the host in adapting to seasonal dietary changes. Here, we compare the gut microbiome composition of wild gray snub-nosed monkeys during winter (from October to December) and spring (from January to March) to understand differences in seasonal nutrient intake patterns. Snub-nosed monkeys are foregut fermenters and consume difficult-to-digest carbohydrates and lichen. To examine the digestive adaptations of gray snub-nosed monkeys, we collected 14 fresh fecal samples for DNA analysis during the winter and spring. Based on 16S rRNA sequencing, metagenomic sequencing, and functional metagenomic analyses, we identified that Firmicutes, Actinobacteria, Verrucomicrobia, and Bacteroidetes constitute a keystone bacterial group in the gut microbiota during winter and spring and are responsible for degrading cellulose. Moreover, the transition in dietary composition from winter to spring was accompanied by changes in gut microbiota composition, demonstrating adaptive responses to varying food sources and availability. In winter, the bacterial species of the genera *Streptococcus* were found in higher abundance. At the functional level, these bacteria are involved in fructose and mannose metabolism and galactose metabolism c-related pathways, which facilitate the breakdown of glycogen, starch, and fiber found in fruits, seeds, and mature leaves. During spring, there was an increased abundance of bacteria species from the *Prevotella* and *Lactobacillus* genera, which aid the digestion of protein-rich buds. Combined, these findings reveal how the gut microbiota adjusts to fluctuations in energy balance and nutrient intake across different seasons in this critically endangered species. Moreover, we also identified *Pseudomonas* in two samples; the presence of potential pathogens within the gut could pose a risk to other troop members. Our findings highlight the necessity of a conservation plan that focuses on protecting vegetation and implementing measures to prevent disease transmission for this critically endangered species.

## 1. Introduction

The growth and reproduction of animals depend on obtaining sufficient nutrition [1,2]. However, food availability and nutritional composition exhibit temporal and spatial fluctuations due to geographical differences as well as changes in climate and ecological conditions. Evolution, hence, favored different behavioral and physiological adaptations to acquire nutrients, including shifting dietary preferences, altered activity patterns, and digestive tract structure [3,4,5,6]. Over the last twenty years, research has increasingly highlighted the fundamental significance of the gut microbiome in terms of nutrition intake, energy extraction, and metabolism [3,7,8,9,10,11,12]. This is particularly evident in plant-eating mammals, whose gut microbiome plays a crucial role in breaking down and fermenting plant structural carbohydrates, facilitating the production of short-chain fatty acids (SCFAs) that can then be utilized by the host as an energy source [13,14,15].

In response to seasonal variations in food availability, plant-eating mammals experience changes in microbiome composition, enabling individuals to adjust digestive efficiency. For example, in summer and fall, when giant pandas (*Ailuropoda melanoleuca*) rely on bamboo leaves as a food source, which contain high amounts of fiber, bacterial species exhibited an overrepresentation of genes associated with the breakdown of raw fiber and the regulation of the cell cycle. However, during periods when they consume bamboo shoots, which contain high amounts of protein, the functional capacity of the gut microbiome expands to include prokaryotic secretion and signal transduction activity [16,17]. Likewise, geladas (*Theropithecus gelada*) also experience microbial changes with seasonal variations in food sources and dietary compositions. In periods with more rainfall, the gut is dominated by cellulolytic and fermentative bacteria specialized in digesting grass. Conversely, in drier periods, bacteria that break down starches found in underground plant parts become dominant [18]. In addition, in their cold, high-altitude habitats, yaks (*Bos mutus*) have a high-carbohydrate and high-fiber diet in spring, autumn, and winter but switch to a high-protein diet in summer. These dietary changes, similarly, also correspond with changes in the gut microbiome, highlighting the significance of *Akkermansia* in regulating nutritional needs [19]. Combined, changes in microbiome composition during different periods enable animals to utilize different nutrients better.

The critically endangered gray snub-nosed monkey (*Rhinopithecus brelichi*) population is restricted in their distribution to a small area of fragmented forest within the Fanjinshan National Reserve, Guizhou Province, China. This last remaining gray snub-nosed monkey population ranges at an altitude of 1400–2100 m across an area of 28 km^2^ of evergreen and deciduous broadleaved forest in the reserve [20]. Like other colobine monkeys, gray snub-nosed monkeys are characterized by a highly specialized and elongated, sacculated stomach that maintains a pH of 6–7 and a highly diverse microbiota [21,22]. The microbiota of the colobine species plays crucial roles in converting cellulose into short-chain fatty acids and facilitating host energy acquisition [23]. Previous field studies demonstrate that the diet of gray snub-nosed monkeys varies seasonally. During winter (October to December) and spring (January to March), especially, they switch from a diverse diet, composed of various food sources, to a specialized diet of consuming buds [24,25]. In winter, when the deciduous tree species shed their leaves, they rely on magnolia buds (32.92%), along with the seeds, fruits, and mature leaves of the evergreen tree species. In spring, when plants begin to sprout new shoots, they feed mainly on plant buds (89.95%) and leaves [24,25]. 

However, little is known about the adaptive responses of gut microbes to the seasonal variation in food source and availability. To date, only Hale et al studied the gut microbiome of gray snub-nosed monkeys [26]. They extracted DNA of the gut microbiota from the feces of seven wild individuals and eight captive individuals in April and July and analyzed the relative abundance of bacteria in the Lachnospiraceae and Ruminococcaceae families using 16S rRNA sequencing technology. The bacteria in the Lachnospiraceae and Ruminococcaceae families live in symbiosis with herbivorous hosts, which are specialized in digesting complex plant materials and producing butyrate [27]. The studied captive gray snub-nosed monkeys exhibited increased proportions of the *Bacteroides* species within their gut microbiota. These *Bacteroides* species are involved in the breakdown of simple sugars and carbohydrates, such as those commonly found in fruits and cornmeal. Hence, dietary variation contributes to disparities in the diversity and functionality of the gut microbiota in gray snub-nosed monkeys [26]. 

It is unclear whether the gut microbiome of gray snub-nosed monkeys exhibits changes in composition in response to seasonal variations in food source and availability. Identifying seasonal differences in gut bacterial diversity may shed light on our understanding of seasonal nutrient intake patterns in gray snub-nosed monkeys and guide conservation efforts to conserve this critically endangered species. To accomplish this, we employed amplicon sequencing, targeting specific regions of 16S rRNA genes and metagenomics, and assessed the presence of pathogenic bacteria. With the suitable habitat for gray snub-nosed monkeys significantly diminishing by more than 90% in recent decades, and their population size dwindling to fewer than 400 individuals [20], monitoring the well-being of this critically endangered primate population becomes crucial. 

## 2. Materials and Methods

### 2.1. Samples Collection and DNA Extraction

Between December 2020 and February 2021, we collected 14 fresh fecal samples from gray snub-nosed monkeys inhabiting the Yangaoping district in the northeastern sector of the Fanjingshan National Reserve. To ensure the freshness of the fecal samples, we assessed their size and color, confirming that they had been voided within the past 24 h. The collected samples were categorized into two groups, the fecal samples obtained in December were designated winter samples, while those collected in January and February were labeled as spring samples. To avoid contaminating the fecal samples, we used sterilized tweezers and petri dishes during sample collection. To avoid sample degeneration, the collected samples were stored in liquid nitrogen and preserved with dry ice before being shipped to the laboratory for cryogenic storage. We stored the samples at −80 °C for no longer than one week before DNA extraction.

We used the QIAamp Fast DNA Stool Mini Kit (Hilden, Germany) to extract DNA from fecal samples (100 to 150 mg) following the manufacturer’s instructions. The extracted DNA underwent 0.8% agarose gel electrophoresis and Green View nucleic acid dye staining. Then, we used the UV transilluminator, specifically a Nanodrop spectrophotometer, to measure the estimated concentration and assess the purity of each sample by recording the 260/280 and 260/230 ratios. To determine the DNA concentration precisely, we employed the Qubit 3.0 fluorescence quantifier for DNA isolation and quantification.

### 2.2. 16S rRNA-Targeted Meta Genomic Sequencing

We employed a 16S rRNA gene sequencing methodology and the V3-V4 region of the 16S rRNA gene was amplified using primers (F: 5′-ACTCCTACGGGAGGCAGCA-3′ and R: 5′-GGACTACHVGGGTWTCTAAT-3′). The PCR process involved three steps. The first step involved an initial denaturation, heating at 98 °C for 1 min. The second step comprised a cycling stage, where the DNA was denatured at 98 °C for 10 s, followed by annealing at 50 °C for 30 s, and then extension at 72 °C for 30 s, repeated for 30 cycles. The third step was a final extension, maintained at 72 °C for 5 min. Then, to measure DNA concentration and purity, we combined equal volumes of 1× loading buffer with the sample and then applied the mixture for electrophoresis on a 2% agarose gel.

Utilizing the Qiagen Gel Extraction Kit, the PCR products were subsequently purified. After the purification, we employed the TruSeq Nano DNA LT Library Prep Kit (Illumina, Foster City, CA, USA) to generate sequencing libraries, followed by evaluation utilizing the Qubit 3.0 Fluorometer and Agilent Bioanalyzer 2100 system. Lastly, the libraries were sequenced on the Illumina NovaSeq platform using the NovaSeq 6000 SP Reagent Kit, with a sequencing run of 500 cycles.

### 2.3. Metagenomic Shotgun Sequencing

A Whole Genome Shotgun (WGS) strategy was applied using the Illumina NovaSeq high-throughput sequencing platform to evaluate the complete DNA of the microbial community. The total DNA was randomly fragmented into short fragments, and 2 × 150 bp paired-end (PE) sequencing libraries were constructed using the Illumina TruSeq Nano DNA LT Library Preparation Kit on the Illumina NovaSeq platform. Quality assessment of the raw data, including base quality distribution, average sequence quality distribution, base composition distribution, and GC content distribution, was performed using FastQC [28]. The raw paired-end sequence data obtained from high-throughput sequencing underwent a series of quality filtering steps. These steps included removing reads with a high proportion of low-quality bases, eliminating reads with a certain proportion of N bases, and excluding reads with overlaps surpassing a specific threshold with adapters. It was important to consider the potential presence of host contamination in the samples. Therefore, the clean data was subjected to BLAST against the host database to identify and filter out reads that could potentially originate from the host [29,30,31]. 

### 2.4. 16S rRNA Gene Compositional Analysis

To ensure enhanced precision and dependability in our analyses and findings, we commenced by eliminating the barcodes and primers. Subsequently, we utilized the FLASH software (V 1.2.7) to merge the R1 and R2 sequence data. Implementing specific filtering parameters, we conducted quality filtering on the raw tags that yielded meticulously filtered tags of exceptional quality. Leveraging the classify-sklearn algorithm in QIIME2 (V1.9.1) [32,33], we employed a pre-trained Naive Bayes classifier to annotate each amplicon sequence variant (ASV) at the species level. By utilizing the ASV annotation outcomes and sample feature tables, we created comprehensive species profiles with all taxonomic levels. Furthermore, we visualized the ASV numbers and classification status at each taxonomic level using R, presenting them as bar plots. Rarefaction curves, Alpha diversity, and Beta diversity indices [34,35] were evaluated for ASVs from groups A and B, and the results were visualized using an R-package ‘ggplot2’. Principal Coordinates Analysis (PCoA) and UPGMA trees were generated using QIIME2 (V1.9.1) to assess species composition differences between groups. To effectively compare the disparities in species abundance distribution between the groups, we employed the ‘pheatmap’ R-package to create a heatmap, which visually depicted the trends. To identify statistically significant variations in gut microbial features across the groups, we conducted LEfSe analyses [36].

### 2.5. Whole Shotgun Metagenomics Analysis

We employed the MEGAHIT software (V 1.2.9) to analyze the clean data assembly [37]. The minimap2 software (V 2.2.0) was employed to align each sample’s quality-filtered effective sequences with their respective contig sequence sets and identify the unmatched sequences. The unmatched sequences were merged, followed by a secondary assembly using the MMseqs2 software (V 13.4511) in linclust mode. This involved removing redundancy from the contig sequence set based on 95% similarity and 90% coverage of the alignment region. The non-redundant contig set was obtained, and the assembled contigs were evaluated for assembly performance. 

To predict Open Reading Frames (ORFs) for Scaftigs (with a length of at least 500 bp) in each sample, we utilized the MetaGeneMark software (V 4.5.3) [38,39]. Information less than 100 nt in length from the prediction results were filtered out [38,40,41]. The ORF prediction results were subjected to the MMseqs2 software [42] for redundancy elimination and the generation of a non-redundant initial gene catalog. For further protein sequence species annotation, the MMseqs2 software in taxonomy mode aligned the non-redundant protein sequences with the NCBI nr database (v2021.10.11). We utilized the alignment result with the highest alignment score to select the species information associated with each protein gene sequence. The alignment result provided the species information linked to that specific protein gene sequence.

To obtain the species annotation information of contig sequences at various taxonomic levels, we selected alignment results with e-values less than or equal to the minimum e-value multiplied by 10. The Blast2lca software, (V 1.0.0) employing the LCA algorithm, was utilized for this purpose. We performed functional predictions based on the KEGG (Kyoto Encyclopedia of Genes and Genomes) and CAZy (Carbohydrate-Activeen ZYmes) databases. We conducted LEfSe analyses to identify statistically significant differences in gut microbial functions between groups. Finally, we performed inter-group differential analysis of functional units KO (KEGG orthology) and predicted KEGG metabolic pathways. Colors were assigned to distinguish upregulated and downregulated KOs, demonstrating variations in functional composition between samples.

## 3. Results

### 3.1. Composition of Gut Microbiota 

We collected seven fecal samples of gray snub-nosed monkeys during winter and seven samples during the spring (Table S1 in Supporting Information). A sum of 1,075,876 high-quality sequences was acquired from the fecal DNA samples, averaging around 76,848 sequences per sample through PE sequencing of the 16S rRNA gene (Table S2 in Supporting Information). These sequences were classified into 4193 ASVs (amplicon sequence variants), averaging 299 ASVs per sample (Table S2 in Supporting Information). The rarefaction curve demonstrated a significant increase in the number of newly discovered microbial species as sequencing depth increased. Once the sequencing data exceeded 5000 sequences, the curve reached a plateau, indicating sufficient sequencing depth to ensure the reliability of subsequent data analysis (Figure S1 in Supporting Information). 

**Figure 1:**
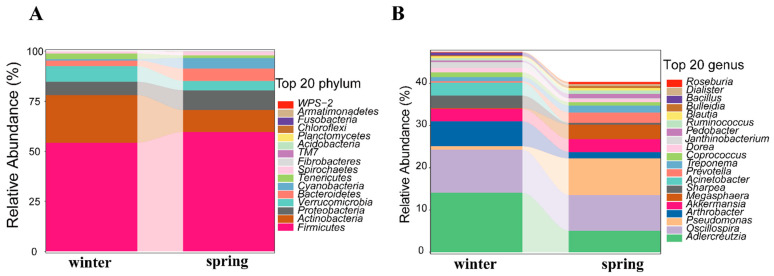
Stacked column of relative abundance of gut microbiota. (**A**): Relative abundance of gut microbiota at the top 20 phyla. (**B**): Relative abundance of gut microbiota at the top 20 genera.

Taxonomic analysis categorized these ASVs into 228 genera and 18 phyla. The Firmicutes phylum was found to be the most prevalent among the gut microbiomes at the phylum level. At the genus level, *Adlercreutzia* emerged as the dominant genus within the gut microbiota (Figure S2 in Supporting Information). Additionally, the winter and spring samples were found to have 18 and 16 identified phyla, respectively, with the top 20 phyla being the same during both seasons. At the genus level, the winter samples contained 187 annotated genera, and the spring samples contained 162. The pathogenic bacterial genus *Pseudomonas* was detected in both winter and spring samples. (Figure 1, Figure S3 in Supporting Information).

### 3.2. Seasonal Changes in Gut Microbiota

**Figure 2:**
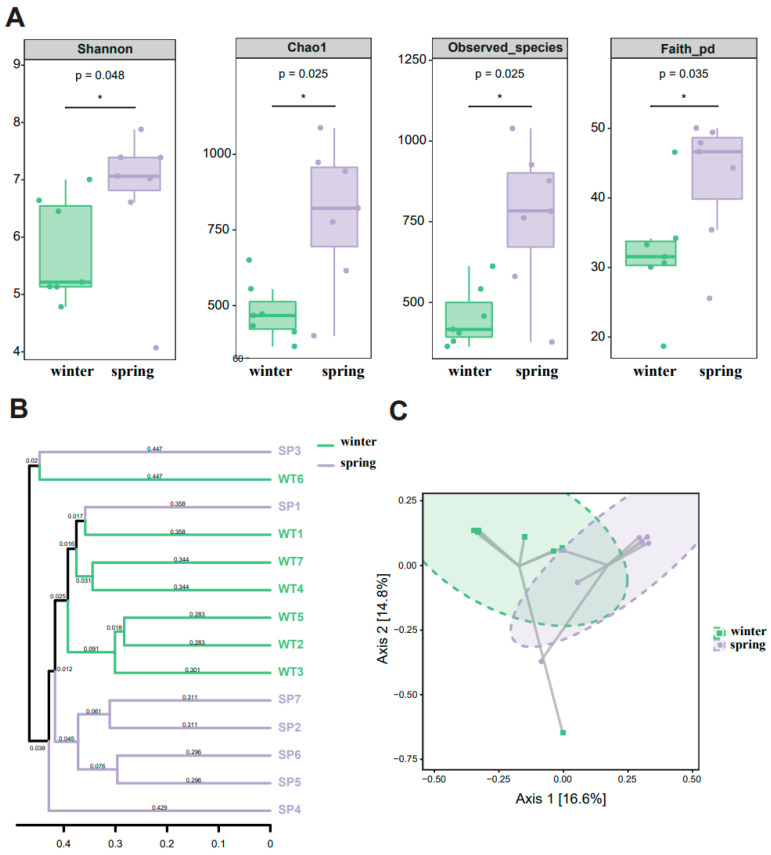
Diversity of gut microbiome across different groups. (**A**): Box plot of Alpha-diversity index differences between winter and spring, * represents a significant difference between winter and spring (*p* < 0.05). (**B**): UPGMA analysis based on Weighted Unifrac distance. (**C**): Cluster analysis result of gut microbiota according to PCoA.

Our findings demonstrate seasonal variations in the gut microbiome of gray snub-nosed monkeys. We observed significant seasonal differences across several alpha diversity indices by applying the Kruskal–Wallis rank-sum test. These included the Chao1 index (*p* = 0.025 < 0.05), the Shannon index (*p* = 0.048 < 0.05), the Observed species index (*p* = 0.025 < 0.05), as well as Faith’s PD index (*p* = 0.035 < 0.05). These results reveal that the gut microbiota of gray snub-nosed monkeys displayed greater diversity during the spring compared to the winter (Figure 2A). In addition, a Principal Coordinates Analysis (PCoA) and an unweighted pair-group method with arithmetic means (UPGMA) clustering analysis revealed that the winter and spring samples formed two distinct clusters with some overlap (*n* = SP1, SP3) spanning both clusters; SP1 and SP3 samples were collected in January. This finding demonstrates a winter-to-spring change in gut microbiota composition (Figure 2B,C). 

**Figure 3:**
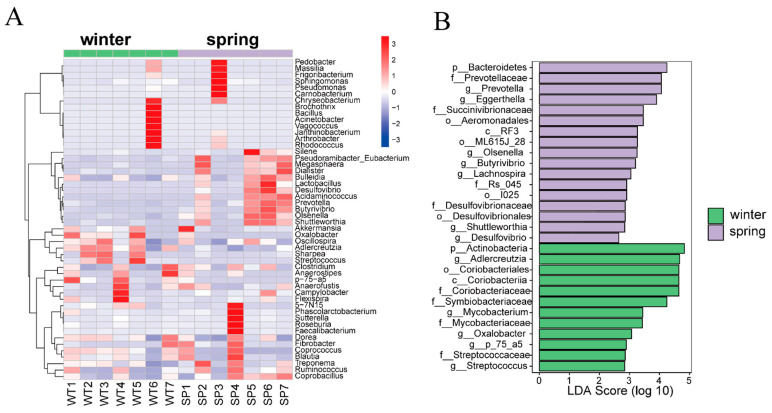
Seasonal differences in gut microbiota of gray snub-nosed monkeys. (**A**): Cluster heat map depicting relative abundance of microbial species during winter and spring, highlighting seasonal shifts in gut microbiota composition. (**B**): Bar chart representing over-represented microbial taxa in winter and spring as identified by LEfSe analysis (*p* < 0.05). Length of each bar is indicative of magnitude of difference in relative abundance between seasons, emphasizing that longer bars correspond to more pronounced seasonal variations. Prefixes (P_, C_, o_, f_, g_) denote taxonomic ranks of Phylum, Class, Order, Family, and Genus, respectively.

In winter, *Streptococcus*, *Oxalobacter*, and *Oscillospira* exhibited significantly higher Z-values, indicating that these bacterial genera were more abundant during the winter. In spring, *Frigoribacterium*, *Lactobacillus*, and *Prevotella*, among others, showed higher Z-values, indicating increased abundance of these bacterial genera during the spring (Figure 3A). Finally, the LEfSe (Linear Discriminant Analysis Effect Size) analysis revealed distinct seasonal patterns in the representation of bacterial genera between the winter and spring stages, indicating significant differences in the microbiome composition across seasons (Figure 3B). In particular, we observed that the relative abundance of the Actinobacteria phylum was higher during the winter season, while the Bacteroidetes phylum showed higher abundance during the spring season (Figure 3B). 

### 3.3. Function of Gut Microbiota in Dietary Fiber Utilization

We generated a total of 633,498,840 high-quality reads from 14 fecal samples of gray snub-nosed monkeys for gut microbial metagenome analysis (Table S4 in Supporting Information). The genome assembly process resulted in an average of 182,190 contigs per sample (Table S5 in Supporting Information). Additionally, we predicted an average of 153,868 genes per sample and annotated approximately 60,003 gene function items using the KEGG database. Using the CAZy database, we identified 361,891 enzymes. We observed a total of 312 pathways, with the most abundant category being metabolism (Figure S4 in Supporting Information). This category encompassed various metabolic processes such as carbohydrate metabolism, amino acid metabolism, energy metabolism, metabolism of cofactors and vitamins, lipid metabolism, and glycan biosynthesis and metabolism (Figure S4 in Supporting Information). We observed a significant enrichment of carbohydrate-active enzymes within the gut microbiota of gray snub-nosed monkeys. Specifically, we found 151,854 families of glycoside hydrolases (GHs), 113,332 families of glycosyl transferases (GTs), 64,430 families of carbohydrate-binding modules (CBMs), 20,148 families of carbohydrate esterases (CEs), 6088 families of polysaccharide lyases (PLs), and 6039 families of auxiliary activities (AAs) (Figure S5 in Supporting Information). Among these, the GT4 gene was the most abundant within the GTs family, followed by CBM50 in the CBMs family, and GH3 and GH23 in the GHs family. These findings suggest that the GHs, GTs, and CBMs families play a crucial role as carbohydrate-active enzymes in the degradation of plant cellulose within the gut microbiota of gray snub-nosed monkeys. 

### 3.4. Seasonal Changes in Gut Microbiome Function

**Figure 4:**
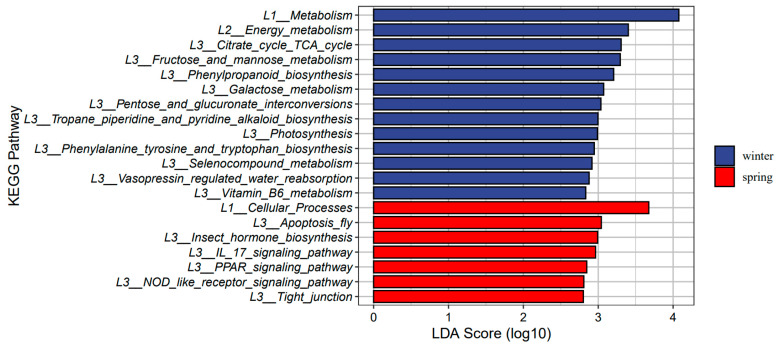
LEfSe analysis identified KEGG pathway enrichment of gut microbiomes in winter and spring (*p* < 0.05). The longer the bar chart, the greater the difference between different pathways. L1_means KEGG pathway’s Level 1 classification; L2_means KEGG pathway’s level 2 classification; and L3_means KEGG pathway’s level 3 classification.

**Figure 5:**
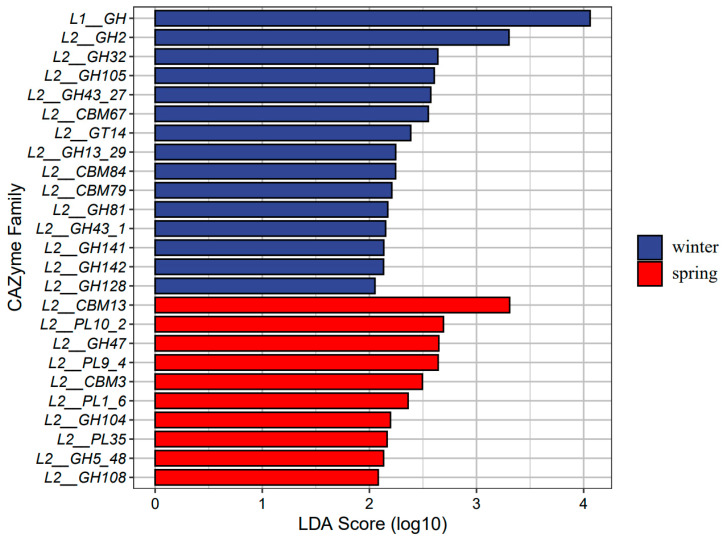
CAZyme family of gut microbiomes identified by LEfSe analysis in winter and spring (*p* < 0.05). The longer the bar chart, the greater the difference between different families. L1_means CAZyme family’s Level 1 classification; L2_means CAZyme family’s level 2 classification; and L3_means CAZyme family’s level 3 classification.

We utilized LEfSe analyses to detect significant seasonal differences in the functional profiles of the microbiomes of gray snub-nosed monkeys between the winter and spring seasons (*p* < 0.05). Analysis using the KEGG database revealed that certain metabolic pathways, including the citrate cycle (TCA cycle), fructose and mannose metabolism, and galactose metabolism, exhibited an over-representation of gene abundance (gene number) during the winter season (Figure 4). In contrast, gene abundance associated with cellular processes such as the IL-17 signaling pathway, PPAR signaling pathway, and NOD-like receptor signaling pathway were over-represented in the spring. The winter samples demonstrated the highest abundance of GH family members (Figure 5). In our investigation, we delved further into the vitamin B6 metabolism pathway, which is primarily associated with immune regulation. Among the 12 differentially expressed genes examined, nine showed upregulation during the winter season, while three genes were upregulated during the spring. Among the 12 KO terms examined, nine were found exclusively in the winter group, while three were unique to the spring group. Our findings indicate that vitamin B6 metabolism may modulate immune responses in gray snub-nosed monkeys, with seasonal variations in gene expression patterns (Figure S6 in Supporting Information).

## 4. Discussion

The symbiotic relationship with gastrointestinal microbes allows mammals to break down plant carbohydrates without genes encoding for cellulose-degrading enzymes [43]. Like other colobines, gray snub-nosed monkeys harbor a diverse microbial population in their elongated and compartmentalized stomach. This microbial community plays a crucial role in fermenting complex carbohydrates like cellulose, hemicellulose, and lignin and breaking down plant secondary compounds. Firmicutes were the dominant phylum in the gut of gray snub-nosed monkeys throughout the winter and spring seasons (Figure S2 in Supporting Information). Firmicutes possess cellulose-degrading capabilities, facilitating the digestion and absorption of structural carbohydrates [23,44]. This finding is consistent with previous studies of the dominant microbial community present in the digestive tract of plant-eating mammals such as the golden snub-nosed monkey (*R. roxellana*), black-and-white snub-nosed monkey (*R. bieti*), cattle (*Bos taurus*), goat (*Capra hircus*), and sika deer (*Cervus nippon*) [45,46,47,48,49]. 

In addition, we also found that Fibrobacteres and Bacteroidetes were prominent phyla in the fecal microbial community of gray snub-nosed monkeys (Figure S2 in Supporting Information). Both *Fibrobacteres* and *Bacteroidetes* participate in cellulose degradation and carbohydrate metabolism [50,51]. The gene functions of these microorganisms were enriched in carbohydrate and amino acid metabolic pathways (Figure S4 in Supporting Information), demonstrating their roles in breaking down plant carbohydrates into short-chain fatty acids that gray snub-nosed monkeys can digest [52]. In addition, given that Fibrobacteres and Bacteroidetes constituted smaller proportions of the gut microbiome than Verrucomicrobia (Figure S2 in Supporting Information), gray snub-nosed monkeys could digest and extract energy and nutrients from carbohydrates and polysaccharides more efficiently than from amino acids [53]. Our findings demonstrate that the gut microbiota of gray snub-nosed monkeys exhibits an enhanced ability to degrade cellulose at the phylum level.

We also observed a seasonal variation in gut microbiota composition. The winter was characterized by a higher number of annotated bacterial genera compared to that of the spring. Conversely, in the analysis of alpha diversity, spring exhibited greater gut microbiota diversity than winter. Alpha diversity is closely related to dietary differences, typically, herbivorous animals have a higher diversity than omnivorous and carnivorous animals [54,55]. This could be attributed to the scarcity of food during winter, leading to a reduced variety of consumed plant species, while the ecological niche of food widens. Notably, there was an elevated abundance of *Streptococcus* in winter. This increase in *Streptococcus* during colder periods is also observed in geladas, which likely enhances the efficient extraction of starch from subterranean food sources [19]. Furthermore, at the functional level, the winter gut microbiota exhibited enrichment in pathways related to fructose and mannose metabolism, as well as galactose metabolism (Figure 4). In winter, when easily digestible food resources become relatively limited, gray snub-nosed monkeys consume a wider variety of food items (buds, fruits, seeds, and mature leaves) compared to spring when they predominantly consume plant buds [24,25]. The improved functionality of these pathways aids the breakdown of glycogen, starch, and fiber found in fruits, seeds, and mature leaves. Moreover, the GH family also improved the digestive efficiency and energy metabolism of gray snub-nosed monkeys during colder periods, given that the GH family was also more abundant during winter than spring (Figure 5) and that GH enzymes degrade plant cell walls and produce a large number of oligosaccharides [56]. These critical microbiotas associated with food digestion could enhance the efficiency of nutrient acquisition for gray snub-nosed monkeys in winter. 

In addition, *Prevotella* and *Lactobacillus* microbes were found to have a higher abundance in spring than in winter (Figure 3A). The genus *Prevotella* is well recognized for its involvement in the degradation of non-cellulosic polysaccharides and pectin, and *Lactobacillus* contributes to protein breakdown [57]. *Prevotella* in the gut microbiota of captive gray snub-nosed monkeys has a greater abundance compared to wild individuals [26]. It has also been noted that there is an increase in *Prevotellaceae* during the summer months, as opposed to other seasons, in the yak’s gut microbiota [19]. Yaks have been found to consume a high-protein diet in summer, and captive gray snub-nosed monkeys tend to have a diet richer in protein compared to their wild counterparts. Based on these observations, our results suggest that the presence of *Prevotella* and *Prevotellaceae* in the gut microbiota of gray snub-nosed monkeys during the spring may indicate a capacity to metabolize a protein-rich diet. Together, our findings demonstrate how the gut microbiota adapts to seasonal variations in nutrient intake and energy demands and contribute to understanding how the gut microbiota adapts to changing dietary and metabolic requirements between winter and spring.

The unique, highly humid seasonal environment of their natural habitat explains the gut microbiome composition and seasonal variation of gray snub-nosed monkeys. Apart from the bacteria that facilitate carbohydrate and protein digestion, we also observed a high abundance of *Actinobacteria*, a genus of bacteria that is known for its anti-inflammatory properties and integral role in maintaining intestinal homeostasis [58]. The abundance of *Arthrobacter* species in the gut of gray snub-nosed monkeys was higher in winter than in spring. In winter, the gut microbiota was significantly enriched in the vitamin B6 pathway (Figure 3B). Given that vitamin B6 has been shown to promote intestinal immune regulation [59], in winter, the gut microbiota of gray-snub-nosed monkeys enhanced resilience against pathogens. This may be due to increased exposure to inflammatory pathogens and bacteria in winter when gray snub-nosed monkeys rely on various food sources such as decaying leaves and roots. Future studies should examine and compare the concentration of inflammatory pathogens and bacteria between different food sources, especially evergreen tree leaves in winter and buds in spring. 

In our study, we observed the presence of the *Pseudomonas* genus in the microbiota of gray snub-nosed monkeys during the winter and spring seasons (WT6 and SP3) (Figure S2 in Supporting Information). It is important to note that *Pseudomonas* includes potential pathogenic species capable of causing infections in various parts of the body [60]. *Pseudomonas* is a genus that can be found in various environments, including moist conditions such as soil and water, where it may be transmitted through wounds [61]. Its presence in the samples may be related to environmental conditions and the behaviors of gray snub-nosed monkeys. Soil consumption has been observed in wild populations [62] and could potentially increase the risk of *Pseudomonas* infection, especially in injured individuals. Furthermore, the higher abundance of *Actinobacteria* in winter might suggest an adaptation to an enhanced immune system during periods of increased digestive effort due to reliance on hard-to-digest food sources. Our results indicate a correlation, but we do not have conclusive evidence to assert a direct causal relationship between the presence of *Pseudomonas*, *Actinobacteria,* and immune system enhancement. Future studies can further investigate the pathogenic potential of species within the *Pseudomonas* and *Actinobacteria* genus in the gray snub-nosed monkey population. 

## 5. Conclusions

In conclusion, our findings demonstrate the seasonal fluctuations in the gut microbiota of gray snub-nosed monkeys. The adaptability of gray snub-nosed monkeys’ microbiome to seasonal variations in nutrient intake improves the ability to adapt to changes in food sources and availability. Moreover, we also identified *Pseudomonas* in two samples; the presence of potential pathogens within the gut could pose a risk to other members of the troop. These findings lay the groundwork for enhancing conservation and management efforts. We propose the following recommendations to safeguard the populations of this critically endangered species: (1) Enhance the conservation of vegetation within gray snub-nosed monkeys’ habitats to ensure an ample supply of plant resources. This will help prevent infections caused by excessive consumption of decaying leaves and roots. (2) Under captive conditions, attention should be paid to the supplementation of high-fiber and high-protein foods while limiting the intake of high-sugar and high-fat items like fruits and nuts. (3) Future research should incorporate analyses that link samples to specific individuals, allowing for a better understanding of the interplay between diet, health, and other individual traits. (4) Future studies should examine a greater number of samples of the intestinal microbiota of gray snub-nosed monkeys for pathogen surveillance and transmission events, specifying likely pathogens and their sources of transmission, such as the entry of dogs and domestic livestock into the reserve, to prevent the introduction of pathogens.

## Data Availability

All sequence data used in this study are available in the GSA database (http://gsa.big.ac.cn/index.jsp accessed on 1 December 2025) under BioProject ID PRJCA025432.

## Supplementary Materials

The following supporting information can be downloaded at https://www.mdpi.com/article/10.3390/ani14131917/s1, Figure S1: Rarefaction curve of 14 samples; Figure S2: The relative abundance of gut microbiota from 14 samples at top 20 phylum; Figure S3: The relative abundance of gut microbiota from 14 samples at top 20 genus; Figure S4: KEGG pathway annotation results; Figure S5: CAZy enzyme annotation results; Figure S6: Vitamin B6 metabolism pathway, red indicates more abundant genes in group A, green indicates more abundant genes in group B; Table S1: Samples information in this study; Table S2: 16S rRNA sequencing statistics of gray snub-nosed monkey title; Table S3: The Numbers of OUT in Phylum taxon; Table S4: Metagenome sequencing statistics of the gut microbiota of gray snub-nosed monkey; Table S5: Metagenome assembly statistics of the gut microbiota of gray snub-nosed monkey.

## References

[1] Dufour D.L., Sauther M.L. (2002). Comparative and evolutionary dimensions of the energetics of human pregnancy and lactation. Am. J. Hum. Biol..

[2] McNab B.K. (2002). The Physiological Ecology of Vertebrates: A View from Energetics.

[3] Van Schaik C.P., Brockman D.K. (2005). Seasonality in primate ecology, reproduction, and life history. Seas. Primates.

[4] Doran D. (1997). Influence of seasonality on activity patterns, feeding behavior, ranging, and grouping patterns in Tai chimpanzees. Am. J. Primatol..

[5] Gursky S. (2000). Effect of seasonality on the behavior of an insectivorous primate, *Tarsius spectrum*. Int. J. Primatol..

[6] Dias P.A.D., Rangel-Negrín A., Canales-Espinosa D. (2011). Effects of lactation on the time-budgets and foraging patterns of female black howlers (*Alouatta pigra*). Am. J. Phys. Anthropol..

[7] Round J.L., Mazmanian S.K. (2009). The gut microbiota shapes intestinal immune responses during health and disease. Nat. Rev. Immunol..

[8] Kau A.L., Ahern P.P., Griffin N.W., Goodman A.L., Gordon J.I. (2011). Human nutrition, the gut microbiome and the immune system. Nature.

[9] Hooper L.V., Littman D.R., Macpherson A.J. (2012). Interactions Between the Microbiota and the Immune System. Science.

[10] Nelson T.M., Rogers T.L., Carlini A.R., Brown M.V. (2013). Diet and phylogeny shape the gut microbiota of A ntarctic seals: A comparison of wild and captive animals. Environ. Microbiol..

[11] Alberdi A., Aizpurua O., Bohmann K., Zepeda-Mendoza M.L., Gilbert M.T.P. (2016). Do Vertebrate Gut Metagenomes Confer Rapid Ecological Adaptation?. Trends Ecol. Evol..

[12] Wei F., Wu Q., Hu Y., Huang G., Nie Y., Yan L. (2019). Conservation metagenomics: A new branch of conservation biology. Sci. China Life Sci..

[13] Flint H.J., Scott K.P., Duncan S.H., Louis P., Forano E. (2012). Microbial degradation of complex carbohydrates in the gut. Gut Microbes.

[14] Bäckhed F. (2011). Programming of host metabolism by the gut microbiota. Ann. Nutr. Metabol..

[15] White B.A., Lamed R., Bayer E.A., Flint H.J. (2014). Biomass utilization by gut microbiomes. Nat. Rev. Microbiol..

[16] Huang G., Wang L., Li J., Hou R., Wang M., Wang Z., Qu Q., Zhou W., Nie Y., Hu Y. (2022). Seasonal shift of the gut microbiome synchronizes host peripheral circadian rhythm for physiological adaptation to a low-fat diet in the giant panda. Cell Rep..

[17] Wu Q., Wang X., Ding Y., Hu Y., Nie Y., Wei W., Ma S., Yan L., Zhu L., Wei F. (2017). Seasonal variation in nutrient utilization shapes gut microbiome structure and function in wild giant pandas. Proc. R. Soc. B Biol. Sci..

[18] Baniel A., Amato K.R., Beehner J.C., Bergman T.J., Mercer A., Perlman R.F., Petrullo L., Reitsema L., Sams S., Lu A. (2021). Seasonal shifts in the gut microbiome indicate plastic responses to diet in wild geladas. Microbiome.

[19] Guo N., Wu Q., Shi F., Niu J., Zhang T., Degen A.A., Fang Q., Ding L., Shang Z., Zhang Z. (2021). Seasonal dynamics of diet–gut microbiota interaction in adaptation of yaks to life at high altitude. Npj Biofilms Microbiomes.

[20] Guo Y.Q., Ren B.P., Dai Q., Zhou J., Garber P.A., Zhou J. (2020). Habitat estimates reveal that there are fewer than 400 Guizhou snub-nosed monkeys, *Rhinopithecus brelichi*, remaining in the wild. Glob. Ecol. Conserv..

[21] Chivers D.J., Hladik C.M. (1980). Morphology of the gastrointestinal tract in primates: Comparisons with other mammals in relation to diet. J. Morphol..

[22] Kay R.F. (1982). On the use of anatomical features to infer foraging behaviour in extinct Primates. Adaptations for Foraging in Nonhuman Primates.

[23] Ley R.E., Hamady M., Lozupone C., Turnbaugh P.J., Ramey R.R., Bircher J.S., Schlegel M.L., Tucker T.A., Schrenzel M.D., Knight R. (2008). Evolution of Mammals and Their Gut Microbes. Science.

[24] Quan G.Q., Xie J.H. (2002). Research on the Golden Monkeys.

[25] Guo Y., Zhou J., Xie J., Garber P.A., Bruford M., Ren B., Li D., Zhou J. (2018). Altitudinal ranging of the Guizhou golden monkey (*Rhinopithecus brelichi*): Patterns of habitat selection and habitat use. Glob. Ecol. Conserv..

[26] Hale V.L., Tan C.L., Niu K., Yang Y., Zhang Q., Knight R., Amato K.R. (2019). Gut microbiota in wild and captive Guizhou snub-nosed monkeys, *Rhinopithecus brelichi*. Am. J. Primatol..

[27] Biddle A., Stewart L., Blanchard J., Leschine S. (2013). Untangling the genetic basis of fibrolytic specialization by Lachnospiraceae and Ruminococcaceaein diverse gut communities. Diversity.

[28] De Sena Brandine G., Smith A.D. (2019). Falco: High-speed FastQC emulation for quality control of sequencing data. F1000Research.

[29] Karlsson F.H., Fåk F., Nookaew I., Tremaroli V., Fagerberg B., Petranovic D., Bäckhed F., Nielsen J. (2012). Symptomatic atherosclerosis is associated with an altered gut metagenome. Nat. Commun..

[30] Karlsson F.H., Tremaroli V., Nookaew I., Bergström G., Behre C.J., Fagerberg B., Nielsen J., Bäckhed F. (2013). Gut metagenome in European women with normal, impaired and diabetic glucose control. Nature.

[31] Scher J.U., Sczesnak A., Longman R.S., Segata N., Ubeda C., Bielski C., Rostron T., Cerundolo V., Pamer E.G., Abramson S.B. (2013). Expansion of intestinal Prevotella copri correlates with enhanced susceptibility to arthritis. eLife.

[32] Bokulich N.A., Kaehler B.D., Rideout J.R., Dillon M., Bolyen E., Knight R., Huttley G.A., Gregory Caporaso J. (2018). Optimizing taxonomic classification of marker-gene amplicon sequences with QIIME 2’s q2-feature-classifier plugin. Microbiome.

[33] Bolyen E., Rideout J.R., Dillon M.R., Bokulich N.A., Abnet C.C., Al-Ghalith G.A., Alexander H., Alm E.J., Arumugam M., Asnicar F. (2019). Reproducible, interactive, scalable and extensible microbiome data science using QIIME 2. Nat. Biotechnol..

[34] Li B., Zhang X., Guo F., Wu W., Zhang T. (2013). Characterization of tetracycline resistant bacterial community in saline activated sludge using batch stress incubation with high-throughput sequencing analysis. Water Res..

[35] Lozupone C.A., Hamady M., Kelley S.T., Knight R. (2007). Quantitative and Qualitative β Diversity Measures Lead to Different Insights into Factors That Structure Microbial Communities. Appl. Environ. Microbiol..

[36] Segata N., Izard J., Waldron L., Gevers D., Miropolsky L., Garrett W.S., Huttenhower C. (2011). Metagenomic biomarker discovery and explanation. Genome Biol..

[37] Nielsen H.B., Almeida M., Juncker A.S., Rasmussen S., Li J., Sunagawa S., Plichta D.R., Gautier L., Pedersen A.G., Le Chatelier E. (2014). Identification and assembly of genomes and genetic elements in complex metagenomic samples without using reference genomes. Nat. Biotechnol..

[38] Mende D.R., Waller A.S., Sunagawa S., Järvelin A.I., Chan M.M., Arumugam M., Raes J., Bork P. (2012). Assessment of Metagenomic Assembly Using Simulated Next Generation Sequencing Data. PLoS ONE.

[39] Qin N., Yang F., Li A., Prifti E., Chen Y., Shao L., Guo J., Le Chatelier E., Yao J., Wu L. (2014). Alterations of the human gut microbiome in liver cirrhosis. Nature.

[40] Sunagawa S., Coelho L.P., Chaffron S., Kultima J.R., Labadie K., Salazar G., Djahanschiri B., Zeller G., Mende D.R., Alberti A. (2015). Structure and function of the global ocean microbiome. Science.

[41] Zhu W., Lomsadze A., Borodovsky M. (2010). Ab initio gene identification in metagenomic sequences. Nucleic Acids Res..

[42] Li W., Godzik A. (2006). Cd-hit: A fast program for clustering and comparing large sets of protein or nucleotide sequences. Bioinformatics.

[43] Dehority B.A. (2002). Gastrointestinal Tracts of Herbivores, Particularly the Ruminant: Anatomy, Physiology and Microbial Digestion of Plants. J. Appl. Anim. Res..

[44] Zhang J., Guo Z., Xue Z., Sun Z., Zhang M., Wang L., Wang G., Wang F., Xu J., Cao H. (2015). A phylo-functional core of gut microbiota in healthy young Chinese cohorts across lifestyles, geography and ethnicities. ISME J..

[45] Costa M.C., Arroyo L.G., Allen-Vercoe E., Stämpfli H.R., Kim P.T., Sturgeon A., Weese J.S. (2012). Comparison of the Fecal Microbiota of Healthy Horses and Horses with Colitis by High Throughput Sequencing of the V3-V5 Region of the 16S rRNA Gene. PLoS ONE.

[46] Kittelmann S., Seedorf H., Walters W.A., Clemente J.C., Knight R., Gordon J.I., Janssen P.H. (2013). Simultaneous Amplicon Sequencing to Explore Co-Occurrence Patterns of Bacterial, Archaeal and Eukaryotic Microorganisms in Rumen Microbial Communities. PLoS ONE.

[47] Oikonomou G., Teixeira A.G.V., Foditsch C., Bicalho M.L., Machado V.S., Bicalho R.C. (2013). Fecal Microbial Diversity in Pre-Weaned Dairy Calves as Described by Pyrosequencing of Metagenomic 16S rDNA. Associations of Faecalibacterium Species with Health and Growth. PLoS ONE.

[48] Omoniyi L.A., Jewell K.A., Isah O.A., Neumann A.P., Onwuka C.F.I., Onagbesan O.M., Suen G. (2014). An analysis of the ruminal bacterial microbiota in West African Dwarf sheep fed grass- and tree-based diets. J. Appl. Microbiol..

[49] Shanks O.C., Kelty C.A., Archibeque S., Jenkins M., Newton R.J., McLellan S.L., Huse S.M., Sogin M.L. (2011). Community Structures of Fecal Bacteria in Cattle from Different Animal Feeding Operations. Appl. Environ. Microbiol..

[50] Jia X., Xi B.D., Li M.X., Yang Y., Wang Y. (2017). Metaproteomics analysis of the functional insights into microbial communities of combined hydrogen and methane production by anaerobic fermentation from reed straw. PLoS ONE.

[51] McCann J.C., Wiley L.M., Forbes T.D., Rouquette F.M., Tedeschi L.O. (2014). Relationship between the Rumen Microbiome and Residual Feed Intake-Efficiency of Brahman Bulls Stocked on Bermudagrass Pastures. PLoS ONE.

[52] Zhou X., Wang B., Pan Q., Zhang J., Kumar S., Sun X., Liu Z., Pan H., Lin Y., Liu G. (2014). Whole-genome sequencing of the snub-nosed monkey provides insights into folivory and evolutionary history. Nat. Genet..

[53] Waters J.L., Ley R.E. (2019). The human gut bacteria *Christensenellaceae* are widespread, heritable, and associated with health. BMC Biol..

[54] Clemente J.C., Pehrsson E.C., Blaser M.J., Sandhu K., Gao Z., Wang B., Magris M., Hidalgo G., Contreras M., Noya-Alarcón Ó. (2015). The microbiome of uncontacted Amerindians. Sci. Adv..

[55] Schnorr S.L., Candela M., Rampelli S., Centanni M., Consolandi C., Basaglia G., Turroni S., Biagi E., Peano C., Severgnini M. (2014). Gut microbiome of the Hadza hunter-gatherers. Nat. Commun..

[56] Bourne Y., Henrissat B. (2001). Glycoside hydrolases and glycosyltransferases: Families and functional modules. Curr. Opin. Struct. Biol..

[57] Chee W.J.Y., Chew S.Y., Than L.T.L. (2020). Vaginal microbiota and the potential of Lactobacillus derivatives in maintaining vaginal health. Microb. Cell Fact..

[58] Binda C., Lopetuso L.R., Rizzatti G., Gibiino G., Cennamo V., Gasbarrini A. (2018). Actinobacteria: A relevant minority for the maintenance of gut homeostasis. Dig. Liver Dis..

[59] Uebanso T., Shimohata T., Mawatari K., Takahashi A. (2020). Functional Roles of B-Vitamins in the Gut and Gut Microbiome. Mol. Nutr. Food Res..

[60] Palleroni N.J. (2010). The Pseudomonas story. Environ. Microbiol..

[61] Spiers A.J., Buckling A., Rainey P.B. (2000). The causes of Pseudomonas diversity. Microbiology.

[62] Yang Y.Q., Lei X.P., Yang C.D. (2002). Fanjingshan Research, Ecology of the Wild Guizhou Snub-Nosed Monkey.

