# Supplementary material for: The Conservation Implications of the Gut Microbiome for Protecting the Critically Endangered Gray Snub-Nosed Monkey (Rhinopithecus brelichi)
Source: Animals (Basel). 2024 Jun 28;14(13):1917. doi: 10.3390/ani14131917 (PMC11240530; doi:10.3390/ani14131917)
Supplement: Supplementary file 1 [file animals-14-01917-s001.zip › animals-3053544-supplementary.pdf]

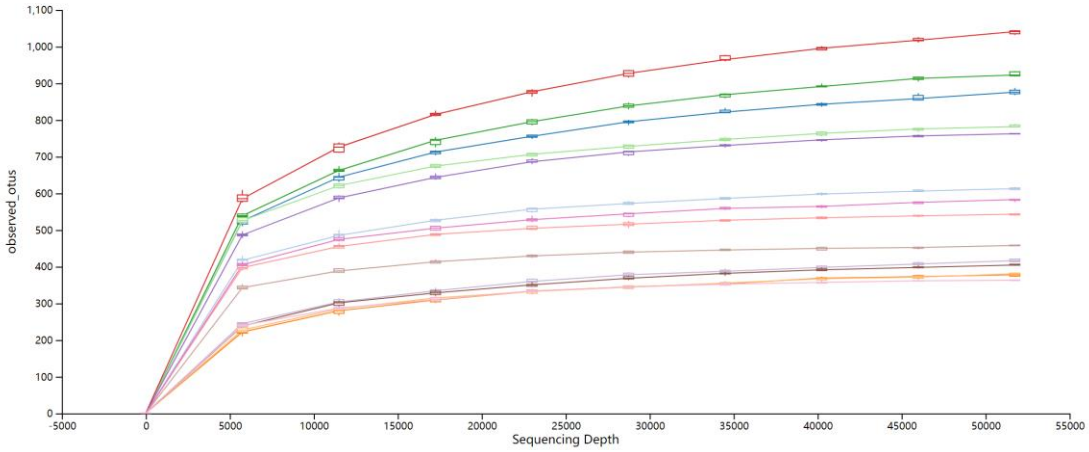

Figure S1 Rarefaction curve of 14 samples

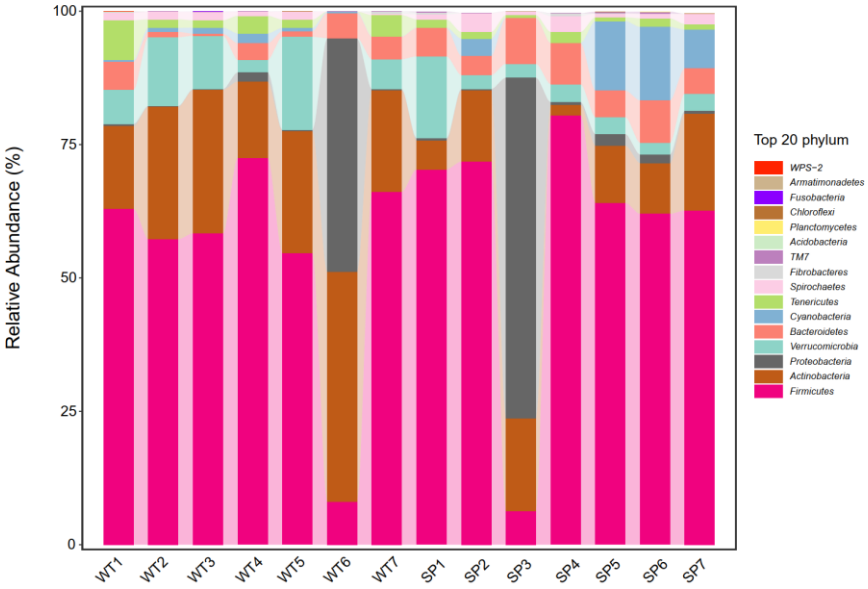

Figure S2 The relative abundance of gut microbiota from 14 samples at top 20 phylum

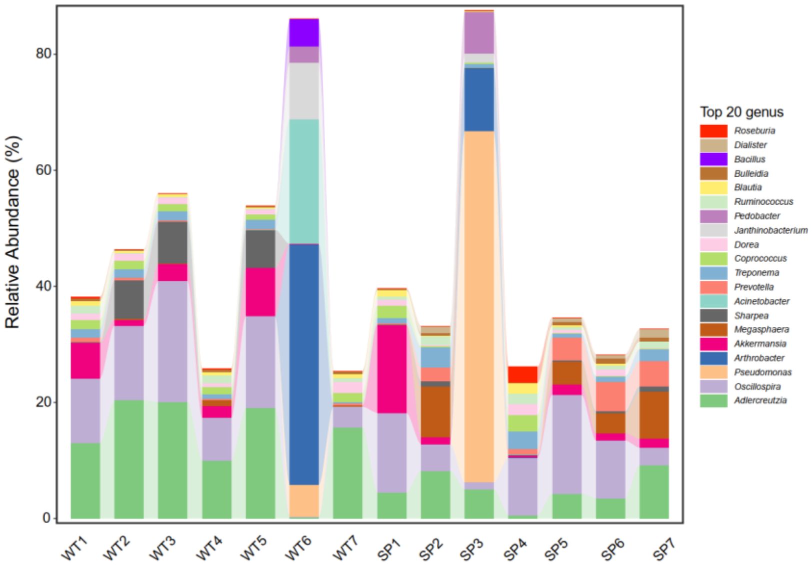

Figure S3 The relative abundance of gut microbiota from 14 samples at top 20 genus

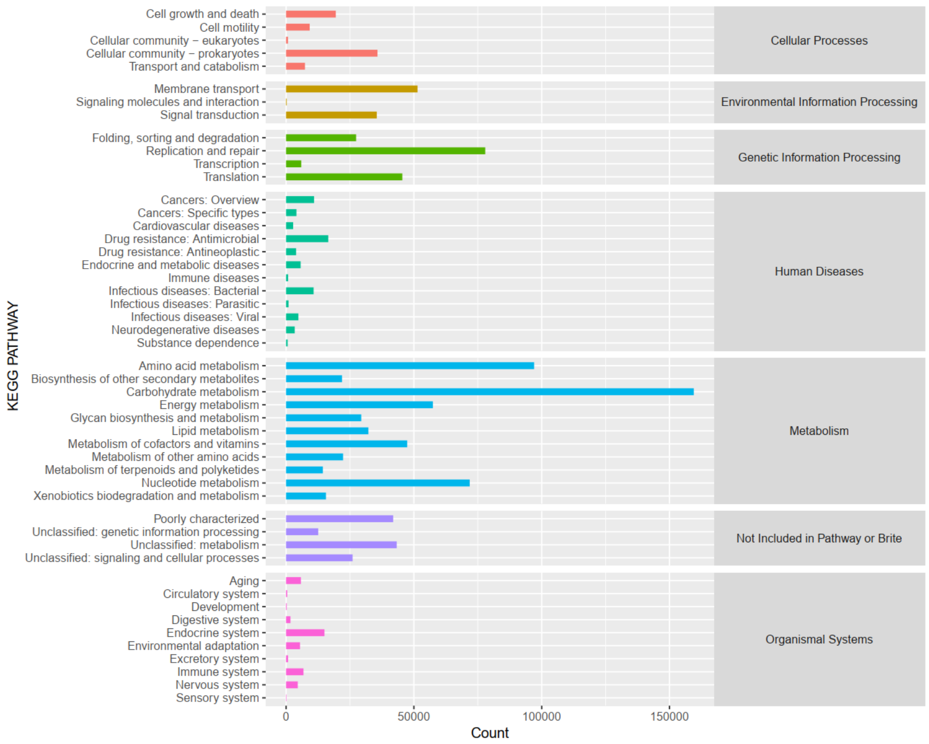

Figure S4 KEGG pathway annotation results

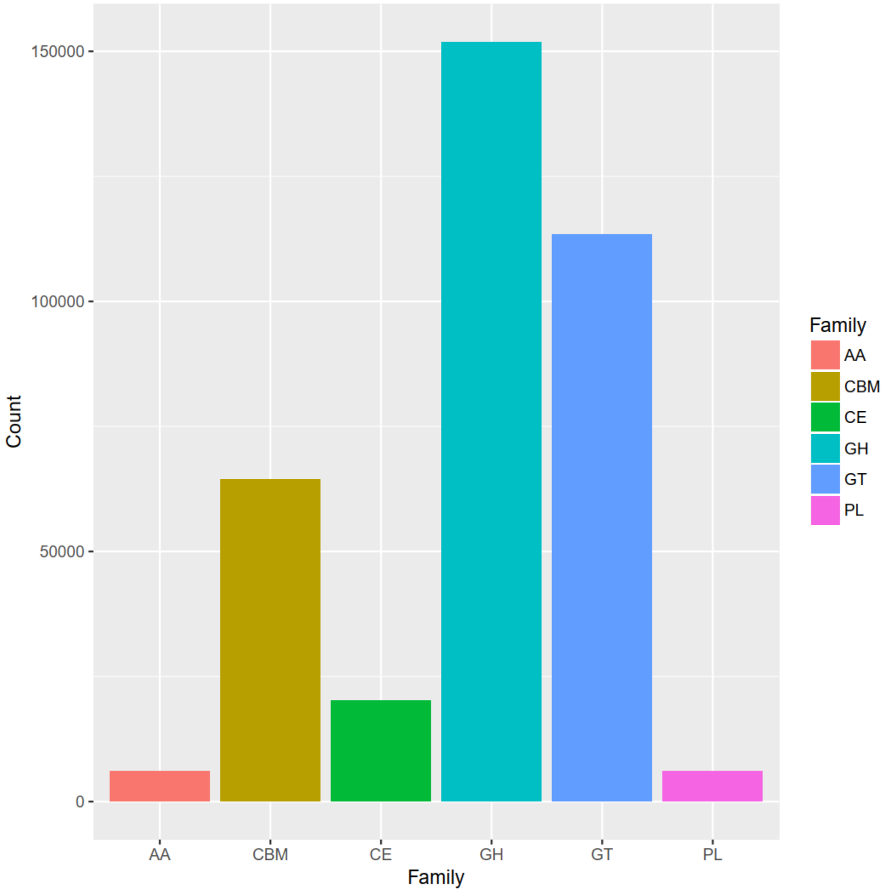

Figure S5 CAZy enzyme annotation results

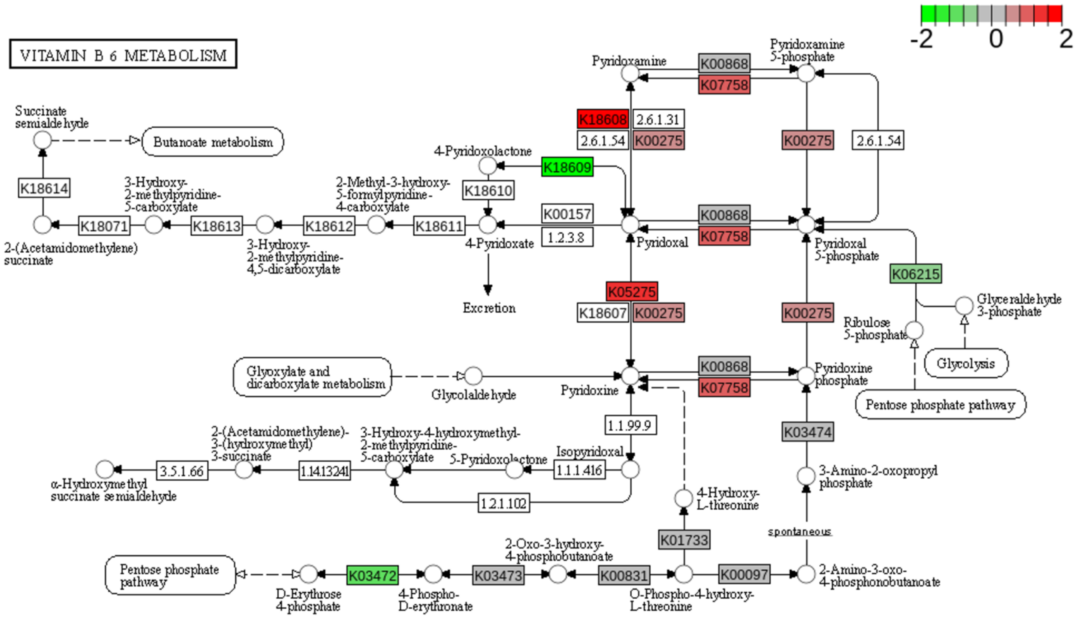

Figure S6 Vitamin B6 metabolism pathway, red indicates more abundant genes in group A, green indicates more abundant genes in group B.

Table S1 Samples information in this study

Sample ID	Species	Age	Sampling position	Sampling season
WT1	gray snub-nosed monkey	adult	Guizhou Fanjinshan	winter
WT2	gray snub-nosed monkey	adult	Guizhou Fanjinshan	winter
WT3	gray snub-nosed monkey	adult	Guizhou Fanjinshan	winter
WT4	gray snub-nosed monkey	adult	Guizhou Fanjinshan	winter
WT5	gray snub-nosed monkey	adult	Guizhou Fanjinshan	winter
WT6	gray snub-nosed monkey	adult	Guizhou Fanjinshan	winter
WT7	gray snub-nosed monkey	adult	Guizhou Fanjinshan	winter
SP1	gray snub-nosed monkey	adult	Guizhou Fanjinshan	spring
SP2	gray snub-nosed monkey	adult	Guizhou Fanjinshan	spring
SP3	gray snub-nosed monkey	adult	Guizhou Fanjinshan	spring
SP4	gray snub-nosed monkey	adult	Guizhou Fanjinshan	spring
SP5	gray snub-nosed monkey	adult	Guizhou Fanjinshan	spring
SP6	gray snub-nosed monkey	adult	Guizhou Fanjinshan	spring
SP7	gray snub-nosed monkey	adult	Guizhou Fanjinshan	spring

Table S2 16S rRNA sequencing statistics of gray snub-nosed monkey

Sample ID	Species	Clean reads	#OTU
WT1	gray snub-nosed monkey	87447	641
WT2	gray snub-nosed monkey	78578	399
WT3	gray snub-nosed monkey	74812	421
WT4	gray snub-nosed monkey	62478	548
WT5	gray snub-nosed monkey	116313	460
WT6	gray snub-nosed monkey	59995	365
WT7	gray snub-nosed monkey	63813	462
SP1	gray snub-nosed monkey	79279	600
SP2	gray snub-nosed monkey	79945	918
SP3	gray snub-nosed monkey	108844	397
SP4	gray snub-nosed monkey	74780	1071
SP5	gray snub-nosed monkey	70827	955
SP6	gray snub-nosed monkey	64144	799
SP7	gray snub-nosed monkey	54621	766

Table S3 The Numbers of OUT in Phylum taxon

Taxon	WT1	WT2	WT3	WT4	WT5	WT6	WT7	SP1	SP2	SP3	SP4	SP5	SP6	SP7
d__Bacteria;p__Acidobacteria	6	0	0	0	0	0	0	0	0	0	0	0	0	6
d__Bacteria;p__Actinobacteria	8014	12832	13936	7396	11817	22318	9867	2846	6913	9019	977	5568	4924	9405
d__Bacteria;p__Armatimonadetes	3	0	0	0	0	0	0	0	0	1	0	0	0	0
d__Bacteria;p__Bacteroidetes	2747	517	203	1605	538	2455	2188	2771	1864	4452	3984	2577	4101	2462
d__Bacteria;p__Chloroflexi	2	0	0	0	1	0	0	0	0	0	0	2	0	1
d__Bacteria;p__Cyanobacteria	166	387	583	953	321	115	40	19	1686	34	20	6735	7125	3723
d__Bacteria;p__Fibrobacteres	23	0	0	0	8	0	229	203	36	1	256	9	8	0
d__Bacteria;p__Firmicutes	32568	29634	30191	37526	28284	4154	34225	36400	37155	3239	41667	33155	32090	32409
d__Bacteria;p__Fusobacteria	0	0	5	0	0	0	0	0	0	0	0	0	0	0
d__Bacteria;p__Planctomycetes	8	0	0	0	0	0	0	0	0	0	0	0	2	0
d__Bacteria;p__Proteobacteria	222	78	54	881	113	22619	67	174	128	33031	279	1107	830	257
d__Bacteria;p__Spirochaetes	767	749	846	398	787	0	135	483	1825	348	1544	366	442	990
d__Bacteria;p__TM7	48	13	7	22	11	5	7	80	7	16	31	158	201	6
d__Bacteria;p__Tenericutes	3836	832	708	1711	801	10	2052	799	626	237	1061	366	837	550
d__Bacteria;p__Verrucomicrobia	3307	6670	5178	1224	9047	38	2924	7939	1349	1357	1741	1642	1141	1686
d__Bacteria;p__WPS-2	2	0	0	0	0	0	0	0	0	0	0	0	0	0
d__Bacteria;p__unclassified_Bacteria	14	15	20	18	9	27	7	19	29	6	70	29	9	75
d__Bacteria;p__unidentified_Bacteria	8	14	10	7	4	0	0	8	123	0	111	27	31	171

Table S4 Metagenome sequencing statistics of the gut microbiota of gray snub-nosed monkey

**Sample ID**	**Number of Reads**	**Number of bases(bp)**	**N (%)**	**GC (%)**	**Q20 (%)**	**Q30 (%)**
S210106B	49327972	7448523772	0.00038	51.5	98.47	95.28
S210112B	44594652	6733792452	0.00038	50.55	98.48	95.28
S210202D	44134322	6664282622	0.00038	43.2	98.25	94.59
S210122D	42844072	6469454872	0.00038	54.22	98.24	94.76
S210201A	44875012	6776126812	0.00038	44.04	98.26	94.63
S210225A	44047148	6651119348	0.00039	45.77	98.3	94.73
S210218A	40942838	6182368538	0.00038	48	98.48	95.17
S210222A	42577446	6429194346	0.00039	49.93	98.3	94.77
S201229B	41719018	6299571718	0.00038	45.21	98.36	94.8
S210109A	52590268	7941130468	0.00038	52.69	98.28	94.89
S210118E	46684744	7049396344	0.00038	51.37	98.38	95.14
S210116A	52828604	7977119204	0.00038	51.23	98.29	94.82
S210117A	42492684	6416395284	0.00038	45.59	98.1	94.15
S210124B	43840060	6619849060	0.00038	57.42	98.29	94.97

Table S5 Metagenome assembly statistics of the gut microbiota of gray snub-nosed monkey

	Min sequence length	Max sequence length	Total sequence number	N20	N50	N90	Total sequence length	GC Number	GC content	Sequences greater than 1kb
WT1	200	493700	187569	17615	2100	482	240576281	116625326	0.484775	45748
WT2	200	346186	245997	7501	1480	467	274749223	141555831	0.515218	58836
WT3	200	174109	274982	4056	956	428	241956334	109518593	0.452638	46113
WT4	200	335911	130945	22244	2360	482	174367686	91909349	0.527101	31886
WT5	200	314111	282395	3855	960	427	247466836	113201937	0.457443	47853
WT6	200	148753	240172	4279	1036	432	219065511	102289388	0.466935	44010
WT7	200	361701	266121	11961	1695	477	318542718	151507903	0.475628	65933
SP1	200	529195	292466	8528	1394	459	317109795	158665121	0.500348	63397
SP2	200	287493	195049	9426	1450	462	216017313	98331523	0.455202	43132
SP3	200	316837	161853	12018	2000	463	197612733	98187883	0.49687	38665
SP4	200	287492	151452	8239	1529	469	172232524	84818000	0.492462	39105
SP5	200	592574	133918	21844	2116	463	166752617	78158651	0.46871	28554
SP6	200	521803	216823	16636	1691	463	253760511	114351175	0.450626	44948
SP7	200	866789	221619	4022	1262	455	224763105	124627199	0.554482	54333
